# Editorial: New strategies for reversing cancer therapy resistance

**DOI:** 10.3389/fphar.2024.1411519

**Published:** 2024-04-18

**Authors:** Yue Du, Xiujun Liu, Lulu Wang, Shengxi Chen

**Affiliations:** ^1^ Department of Pharmacy, The First Affiliated Hospital of Zhengzhou University, Zhengzhou, China; ^2^ Chinese Academy of Medical Sciences and Peking Union Medical College, Beijing, China; ^3^ Biodesign Center for Bioenergetics, Arizona State University, Tempe, AZ, United States

**Keywords:** drug resistance, cancer therapy, mechanism, reverse strategy, novel drug

Cancer stands as one of the leading causes of mortality worldwide. In the past decade, remarkable achievements in anticancer therapy have been made due to tremendous innovations. However, the emergence of drug resistance remains a major challenge in cancer treatment. Drug resistance can be caused by complex molecular mechanisms such as gene mutations, epigenetic dysregulation, microenvironment alterations, etc., which limits the effectiveness of anticancer therapies, and causes cancer recurrence and metastasis, thus being a major cause of cancer-related death (Housman et al., 2014). Various clinical strategies, including combination therapies and the utilization of epigenetic drugs, have been employed to mitigate or reverse drug resistance with some success (Morel et al., 2020). Nevertheless, the continued progression of cancers in treated patients and the resistance observed in some individuals indicate that current approaches to overcome resistance are far from sufficient, thus further research and innovation are required.

To overcome the challenge, identifying the drug resistance-associated genes is important. Zhang et al. reviewed a genome-wide CRISPR/Cas9 screening method for identifying potential drug resistance-associated genes (Zhang et al. *Genome-wide CRISPR/Cas9 screening for drug resistance in tumors*). This screening approach holds substantial promise for advancing the treatment of malignancies that have developed drug resistance. This article provides an overview of drug resistance pathways such as the KEAP1/Nrf2 pathway, MAPK pathway, NF-κB pathway, etc.

In addition to the gene mutation, numerous studies have discovered the close relationship between lipid metabolism and cancer drug resistance. Wang et al. reviewed the alterations in lipid metabolism associated with drug resistance and elucidate how lipid metabolism influences this resistance (Wang et al. *Lipid metabolism as a target for cancer drug resistance: progress and prospects*). It has been indicated that combination therapy could induce changes in lipid-related metabolic pathways, potentially reversing the progression of cancer drug resistance and augmenting or restoring sensitivity to therapeutic drugs. Thus, this review explores the intersection of medication combination with lipid metabolism and drug resistance, offering novel insights and strategies for future tumor treatment.

Endoplasmic reticulum stress (ERS) represents a cellular response mechanism to counter hypoxia and various stresses. Accumulating evidence suggests that prolonged stress can foster the onset, progression, and drug resistance in tumors through the unfolded protein response. Qing et al. provide an overview of ERS mechanisms and tumor multidrug resistance (MDR), elucidate their interrelationship, and outline the potential of targeting ERS to enhance the therapeutic outcomes (Qing et al. *Crosstalk between endoplasmic reticulum stress and multidrugresistant cancers: hope or frustration*).

Furthermore, two cellular protein biomarkers have been reported in this collection. Wang et al. reported that the nucleolar protein 58 (NOP58) was overexpressed in two 5-fluorouracil (5-FU)-resistant advanced colorectal cancer cell lines, HCT116-5FuR and Lovo-5FuR, coinciding with an increase in aerobic glycolysis rate (Wang et al. *NOP58 induction potentiates chemoresistance of colorectal cancer cells through aerobic glycolysis as evidenced by proteomics analysis*). Knockdown of NOP58 resulted in decreased glycolysis and increased sensitivity of HCT116-5FuR and Lovo-5FuR cells to 5-FU. These findings from proteomic analysis shed light on a novel target implicated in cellular adaptation to 5-FU, offering a potential new therapeutic avenue to combat resistance. The multifunctional molecule ceruloplasmin (CP), involved in iron metabolism, has not been thoroughly investigated regarding its expression pattern, prognostic significance, and association with immune cells in gliomas. Jia et al. revealed a significant association between CP expression and immune pathways in gliomas by analysis of various databases (Jia et al. *Ceruloplasmin is associated with the infiltration of immune cells and acts as a prognostic biomarker in patients suffering from glioma*). This study indicates CP as a potential therapeutic target for gliomas and a predictor of immunotherapy effectiveness.

Breast cancer currently ranks as the most prevalent malignancy with a high mortality rate. A number of target-therapy drugs have been approved by the FDA to treat HER2-positive breast cancer (Cai et al., 2022; Cai et al., 2023). However, prolonged treatment using these drugs often results in drug resistance, complicating subsequent treatment decisions. In a case report, Li et al. used disitamab vedotin (RC48) to treat a female patient with HER2-positive breast cancer who developed drug resistance and disease progression after receiving multiple lines of anti-HER2 targeted therapy (Li et al. *Disitamab Vedotin (RC48) for HER2-positive advanced breast cancer: a case report and literature review*). The tumor exhibited both size reduction and stabilization following treatment with RC48. This case study highlights the potential clinical efficacy of RC48 as a promising therapeutic option for patients resistant to HER2-targeted therapies. Huang et al. reviewed the role of short peptides in breast cancer to overcome the drug resistance (Huang et al. *The Role of Peptides in Reversing Chemoresistance of Breast Cancer: Current Facts and Future Prospects*). In this review, diverse mechanisms through which various peptides reverse drug resistance in breast cancer were described, encompassing the promotion of cancer cell apoptosis, facilitation of non-apoptotic regulatory cell death, inhibition of cancer cell DNA repair mechanisms, alteration of the tumor microenvironment, suppression of drug efflux mechanisms, and enhancement of drug uptake. Deng et al. reviewed a natural product, Ginsenosides as the candidates to treat breast cancer patients (Deng et al. *Updating the therapeutic role of ginsenosides in breast cancer: a bibliometrics study to an in-depth review*). In this review, the various mechanisms of ginsenosides against breast cancer were summarized including apoptosis induction, autophagy stimulation, inhibition of epithelial-mesenchymal transition and metastasis, and regulation of miRNA and lncRNA.

Chronic myeloid leukemia (CML) is a type of myeloproliferative neoplasm triggered by a BCR-ABL fusion gene (Askmyr et al., 2014). The T315I mutation of BCR-ABL is the major cause of resistance to imatinib. Gao et al. investigated the impact of I13, a potent histone deacetylase (HDAC) inhibitor, on differentiation blockage in CML cells (Gao et al. *I13 overrides resistance mediated by the T315I mutation in chronic myeloid leukemia by direct BCR-ABL inhibition*). The results revealed that I13 efficiently depleted BCR-ABL in CML cells expressing the T315I mutation, impeding its function as a scaffold protein that regulates the chronic myeloid leukemia signaling pathway and mediating cell differentiation.

Kanner et al. explored a combination therapy to overcome multidrug resistance (MDR) in cancer cells (Kanner et al. *Cytotoxicity and Reversal Effect of Sertraline, Fluoxetine, and Citalopram on MRP1-and MRP7-mediated MDR*). Three selective serotonin reuptake inhibitor (SSRI) drugs—sertraline, fluoxetine, and citalopram, exhibited inhibitory or reversal effects in conjunction with chemotherapy on both MRP1- and MRP7-overexpressing cells, suggesting their repurposing potential in combating MDR in cancer cells. These findings offer a promising avenue for leveraging FDA-approved medications in combination with therapy protocols to address highly resistant malignancies.

Taken together, this special collection overviews the current development to study the mechanisms of multidrug resistance including the identification of gene mutation, lipid metabolism, endoplasmic reticulum stress involvement, and novel protein markers; and highlights the interesting insights into new and complementary treatments to overcome the drug resistance for cancer treatment.
